# Does transrectal ultrasonography-guided biopsy of the prostate lead to possible further metastasis via circulating tumor cells?

**DOI:** 10.3906/sag-1904-58

**Published:** 2019-12-16

**Authors:** Mustafa Kemal ATİLLA, Bahattin AVCI, Lokman İRKILATA, Mustafa AYDIN, Alper BİTKİN, Mevlüt KELEŞ, İnci YÜCEL, Mahmut ULUBAY

**Affiliations:** 1 Department of Urology, Samsun Research and Training Hospital, Samsun Turkey; 2 Department of Biochemistry, School of Medicine, Ondokuz Mayıs University, Samsun Turkey; 3 Department of Pathology, Samsun Research and Training Hospital, Samsun Turkey

**Keywords:** Prostate, biopsy, circulating tumor cells, metastasis

## Abstract

**Background/aim:**

We evaluate whether transrectal ultrasonography (TRUS)-guided prostate biopsy might lead to spillage of tumor cells into peripheral blood as a result of disruption of the epithelial barrier and ultimately result in metastasis.

**Materials and methods:**

Eighty-eight patients underwent TRUS-guided prostate needle biopsy due to prostate-specific antigen (PSA) increase or abnormal digital rectal examination at the Samsun Research and Training Hospital (Samsun, Turkey) between April 2016 and September 2018. Approximately 10 mL of whole blood was collected from patients before, 1 week after, and 1 month after biopsy. Samples were analyzed for CD117 positivity and prostate-specific membrane antigen (PSMA) levels using flow cytometry. Patients with pathologically determined prostate cancer and without CD117 positivity before biopsy were included in the study. The study group thus consisted of 55 patients.

**Results:**

Subjects’ PSA levels ranged from 2.3 to 40.0 ng/mL (median: 7.9 ng/mL), and their Gleason score was a median of 7 (range: 5–9). PSMA levels ranged between 9.3 ng/mL and 118.5 ng/mL and CD117 antigen levels between 0 and 5. We detected no CD117-positive cells in blood samples collected 7 days or 1 month after biopsy.

**Conclusion:**

We detected no circulating tumor cells in the peripheral circulation following biopsy. Prostate needle biopsy seems to be a safe method in terms of spillage of tumor cells into blood circulation as a possible cause of further metastasis.

## 1. Introduction

Prostate cancer is the most common noncutaneous cancer in men in the United States [1]. Transrectal ultrasonography (TRUS)-guided biopsy of the prostate has been routinely used for the detection of prostatic malignant diseases since being introduced by Hodge et al. in 1989 [2]. In addition to advanced biopsy techniques, prostate-specific antigen (PSA) screening has led to an increase in the early detection of prostate cancer. However, biopsy is still necessary for definite pathological diagnosis. 

Circulating tumor cells (CTCs) circulate in the peripheral blood and are released from the primary tumor or metastatic site. They are thought to play a critical role in the hematogenous spread of malignancy and leading metastases [3]. CD117 (c-kit) is a 145-kDa molecular-weight cell surface antigen and protooncogene. It is a receptor tyrosine kinase located in the structure of the single chain type 1 glycoprotein consisting of 976 amino acids and in the plasma membrane. At the beginning of the 1990s, stem cell factor (SCF) was identified as a c-kit ligand [4]. c-kit encodes a transmembrane tyrosine kinase receptor [5], a member of the platelet-derived growth factor (PDGF) and colony-stimulating factor-1 (CSF-I) receptors. The kinase activity of CD117 has been shown to play a role in the pathophysiology of tumors such as mast cell leukemia, germ cell tumors, gastrointestinal tumors, acute myeloblastic leukemia, neuroblastoma, melanoma, ovarian and breast cancers, and small-cell lung cancer [6]. There is evidence that c-kit and SCF together play a role in various hematological and nonhematological malignancies [7]. Prostate-specific membrane antigen (PSMA) is a type II integral membrane glycoprotein with enzymatic functions that acts as glutamate-preferring carboxypeptidase (glutamate) in the human prostate tissue and that plays a role in folic acid utilization and metabolism [8]. The PSMA protein has a unique three-part structure consisting of an interior of 19 amino acids, a transmembrane part of 24 amino acids, and a 707-amino acid exterior [9]. Although PSMA is present in the prostate secretory-acinar epithelium, it is overexpressed in prostate cancer, which is reflected in elevated blood serum levels [10]. Increased expression has been reported in patients with hematogenous micrometastasis in prostate carcinomas [11]. PSMA expression has been shown in various cancers, including kidney, colon, and breast carcinomas as well as the prostate, in addition to various benign changes [11]. Increased expression has been reported in patients with prostate cancer with hematogenous micrometastases. PSMA expression has also been documented in extraprostatic tissues, including the small bowel and brain [11].

The enumeration of CTCs is regarded as a valuable predictor of possible metastasis and prognosis [12]. CTCs have been found to be useful and may also be prognostic predictors in various stages of prostate cancer, extending from localized disease through metastasis [13]. CTC numbers have been shown to be predictive in the differentiation of unfavorable and favorable groups using discrete cut-off values (≥5 CTCs/7.5 mL of blood vs. ≤4 CTCs) in patients with progressive metastatic breast, colon, or prostate cancer [14].

The purpose of this study was to evaluate whether TRUS-guided prostate biopsy may lead to spillage of tumor cells into peripheral blood as a result of the disruption of the epithelial barrier in terms of its value in predicting future metastasis with the aid of surface biomarkers CD117 and PSMA using flow cytometry. To the best of our knowledge, this is the first prospective study to investigate the correlation between prostate biopsy and CTC numbers via flow cytometric analysis. 

## 2. Materials and methods

Eighty-eight patients admitted to our department due to PSA increase or abnormal digital rectal examination and scheduled for TRUS-guided prostate biopsy between April 2016 and September 2018 were included in the study. Approval for the study was granted by the Ondokuz Mayıs University Ethics Committee, Samsun, Turkey. Detailed written informed consent was obtained from all patients. Approximately 10 mL of whole blood was collected from all patients before biopsy and sent immediately for flow cytometric analysis. All patients then underwent transrectal needle biopsy with 12 cores under TRUS guidance. All specimens were examined by the same pathologist. Cases reported as nonmalignant and patients with metastatic disease were excluded from the study. Cases pathologically determined as prostate cancer and without CD117 positivity prior to biopsy were included in the study. These patients underwent CD117 analysis by flow cytometer 1 week and 1 month after biopsy. Plasma PSMA levels were measured in the same samples at the same time. Before each analysis via flow cytometry, control samples of other origins already present in our laboratory and known to be CD117-positive were studied, and the study samples were analyzed subsequently. Flow cytometric analysis of CD117 lymphocytes isolated from whole blood was performed with density gradient centrifugation using Biocoll separating solution (Biochrom GmBH, Germany) according to the manufacturer’s instructions. Pelleted lymphocytes were resuspended in bovine serum albumin stain buffer (Becton Dickinson, BD Pharmingen, Cat No. 554657). Isolated lymphocytes were incubated with fluorescently conjugated antibodies (monoclonal mouse antihuman CD117, c-kit/APC Clone 104D2, Dako, Denmark).

Samples were analyzed on a FACSCanto II flow cytometer with 2 lasers and 8 colors (Serial No. V87500089, Becton Dickinson, USA). APC was detected using 633-nm and 640-nm filters. A total of 100,000 events were acquired for each specimen, and data were analyzed with FACSDIVA software (BD, USA). Data were expressed as percentages of positive cells with CD117 via flow cytometry.

### 2.1. Analysis of PSMA 

Concentrations of human PSMA in plasma were measured using commercially available enzyme-linked immunosorbent assay (ELISA) kits (Human PSMA ELISA Kit, Cat No. YHB365Hu, Shanghai Yehua Biologic Technology Co. Ltd., Shanghai, China). Enzymatic reactions were quantified on an automatic microplate photometer. PSMA levels were expressed as ng/mL. The mean interassay coefficient of variation (CV) % and intraassay CV % for PSMA were <12% and <10%, respectively. All assays were conducted according to the manufacturer’s instructions. Samples exhibiting higher concentrations were diluted and measured in duplicate. 

Statistical analysis was performed with IBM SPSS 23 software (IBM Corp., Armonk, NY, USA). The normality of data was assessed using the Kolmogorov–Smirnov test. The Spearman correlation and Friedman tests were used to compare data without normal distribution. Correlation tests were used to determine whether correlation existed among age and PSA values and Gleason scores. Data are expressed as median (min–max). P < 0.05 was considered statistically significant.

## 3. Results

Upon pathological examination of TRUS-guided biopsies, 28 (31.8%) of the 88 cases were identified as benign while metastatic disease was detected in five cases using other diagnostic methods (whole-body bone scan). These 33 (37.5%) patients were therefore excluded from the study, which finally involved 55 (62.5%) cases pathologically identified as prostate adenocarcinoma. The patients’ median age was 66.5 years (range: 56–78). The median PSA level was 7.9 ng/mL (range: 2.3–40.0) and median Gleason score was 7 (range: 5–9). We detected a statistically significant positive but weak correlation between patients’ ages and PSA values (P = 0.03, Spearman correlation coefficient r = 0.29). No correlation was observed between age and Gleason scores (P = 0.06, r = 0.26). PSMA levels ranged between 9.3 ng/mL and 118.5 ng/mL and CD117 antigen levels between 0 and 5. No CD117-positive cells were detected in blood samples from patients 7 days or 1 month after biopsy. PSMA data are shown in the Table. The Figure shows the CD117 results from a control specimen and samples from patients prior to biopsy and 7 days and 1 month after biopsy via flow cytometry. 

**Table 1:** PSMA levels.

	Before TRNB	Seven days afterTRNB	One month after TRNB	P
n	55	55	55	
PSMA median (min–max)	20.7 (9.3–117.9)	22.5 (12.1–118.5)	20.5 (13.4–104.0)	0.42

PSMA: Prostate-specific membrane antigen. TRNB: Transrectal needle biopsy.

**Figure:**
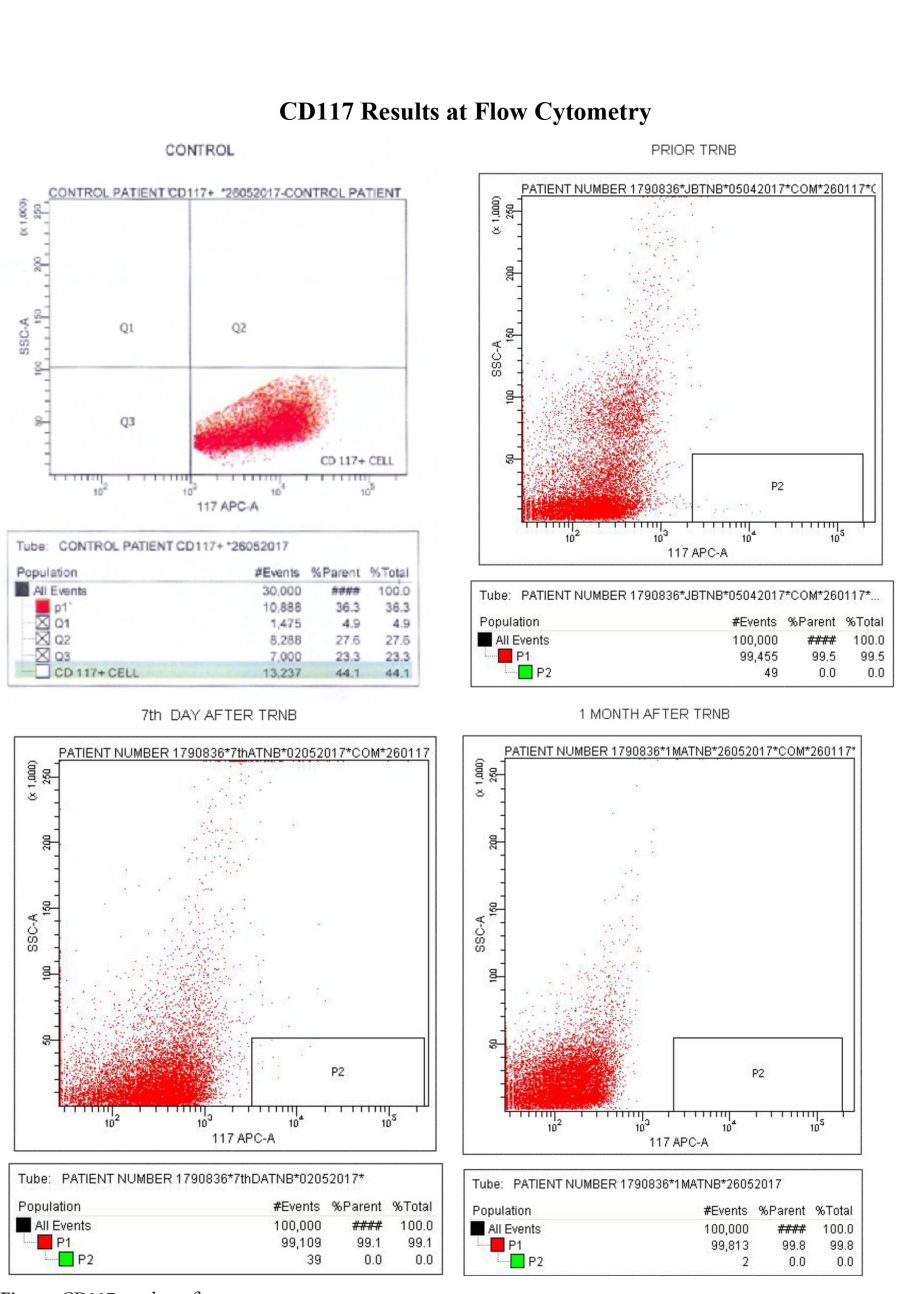
CD117 results at flow cytometry.

These results revealed that no CTCs exceeding the critical level of five cells per 7.5 mL of peripheral blood were observed upon flow cytometric analysis. 

## 4. Discussion

Despite recent advances in PSA-based screening in the form of free and complexed PSA, PSA density, PSA velocity, and age-specific ranges, and imaging methods such as multiparametric magnetic resonance imaging, TRUS-guided prostate biopsy remains the standard technique for routine biopsy as well as for precise identification of malignant tissue. 

Due to some limitations of PSA in the context of pathological stages of the disease, there has recently been considerable interest in CTCs, particularly focusing on different stages of prostate cancer. CTCs in whole-blood samples have been used as an alternative marker in disease determination and prognosis, and in the search for more reliable tests for diagnosing malignant diseases with localized and metastatic phases [15]. Detecting CTCs with high sensitivity and specificity is an important objective of CTC studies in prostate cancer and other solid tumors. Improvements in CTC capture by novel capture antibodies (e.g., mesenchymal antigens), negative selection methods, novel CTC chip designs that enhance CTC yield, and improved CTC molecular profiling technologies will assist further exploration of CTCs and their implications for metastatic prostate cancer. 

The CellSearch System is the first standardized system approved by the Federal Drug Administration for detecting and quantifying CTCs in peripheral blood [16]. Detection by CellSearch depends on EpCAM expression in CTCs, which are subsequently identified as nucleated cells positive for cytokeratin 8, 18, or 19 expression and negative for leukocyte antigen CD45 expression by immunofluorescence staining. This CTC detection technology has been widely used in prostate cancer research. CellSearch results are highly reproducible between laboratories, and the results are stable for samples transported for as long as 72 h [17].

In general, a threshold of 5 cells per 7.5 mL of peripheral blood has been used to estimate prognosis, and cell counts above this level are widely interpreted as a sign of metastatic disease [18]. Allard et al. detected CTCs in 107 of 188 patients with metastatic prostate cancer compared to 1 of 344 healthy male controls [13]. In a study evaluating whether CTCs in peripheral blood samples correlate with tumor volume, pathological stage, and Gleason score in men with localized prostate cancer, Davis et al. observed no correlation between number of CTCs and known prognostic factors in these subjects [19]. Interestingly, their CTC positivity rates were almost identical for patients with prostate cancer (21%) and the healthy controls (20%). In addition, 84% of patients with preliminary positive CTC values were negative by 6 weeks after surgery. CTCs provide real-time and easy access to tumor cells, but there are also limitations to CTC studies. De Bono et al. reported a nondetection rate of CTCs of more than 50% in patients with metastatic castration-resistant prostate cancer [12].

Miyamoto et al. reported that some CTCs may entirely lack epithelial biomarkers [20]. There are several possible explanations for this lack of CTC detection, including loss of rare cells through multiple capture and purification steps, strict characterization definitions, inefficient magnetic separation of labeled cells throughout a large population of unlabeled cells, and other technical issues. 

PSMA is a type II integral membrane glycoprotein that exists in the prostate secretory-acinar epithelium and is highly expressed in prostate cancer. Recent evidence suggests that PSMA is also expressed in the tumor-associated neovasculature [21]. Increased expression has been reported in patients with prostate cancer with hematogenous micro metastases. PSMA expression has also been documented in extraprostatic tissues, including the small bowel and brain [11].

CD117 is a cell surface antigen and protooncogene with a molecular weight of 145 kDa identified as a receptor tyrosine kinase on the plasma membrane in the early 1990s. Activation of tyrosine kinase by somatic mutation has been documented in several malignancies, including gastrointestinal stromal tumor, seminoma, acute myelogenous leukemia, and mastocytosis. A pathophysiological role of this kinase by means of paracrine or autocrine activation has also been postulated in some other malignancies, including small-cell lung and ovarian cancer [6]. Mainetti et al. observed that bone-induced CD117 antigen-positive cells increased the invasion and migration of prostate cancer cells in mice with prostate cancer with bone metastasis [22].

Why did we employ flow cytometry for the analysis and detection of cells? Flow cytometry is considered a reliable method for the detection and analysis of rare CTCs. Bhagwat and Carpenter described flow cytometry as a powerful cell analysis technique that is being increasingly used in this field and that permits easy recovery of viable cells for molecular analysis. They also proposed it as an attractive technology for cancer research and as a diagnostic tool [23].

CTCs can be detected by flow cytometry, which provides sensitive detection of CD117 antigen positivity. To the best of our knowledge, this is the first study evaluating flow cytometric detection of CD117 antigen-positive cells. CD117 antigens were analyzed in circulating cells before and after prostate biopsy in order to establish whether these cells may lead to possible further metastasis. We determined no CD117 antigen-positive cells in blood specimens obtained after biopsy from patients without CD117 antigen positivity before biopsy. These results suggest that no CTC spillage into the peripheral circulation occurred following biopsy. Our hypothesis that TRUS-guided prostate biopsy may lead to spillage of tumor cells into peripheral blood is therefore invalid.

In conclusion, our results reveal that TRUS-guided prostate needle biopsy does not result in clinically significant levels of CTCs in peripheral blood. We therefore conclude that prostate needle biopsy is a safe method in terms of the spillage of tumor cells into the peripheral blood as a result of prostatic epithelial barrier disruption.

## Acknowledgment

Dr. Naci Murat, Assistant Professor of Biostatistics at Ondokuz Mayıs University, Department of Biostatistics, analyzed the data.
